# A broad atlas of somatic hypermutation allows prediction of activation-induced deaminase targets

**DOI:** 10.1084/jem.20171738

**Published:** 2018-03-05

**Authors:** Ángel F. Álvarez-Prado, Pablo Pérez-Durán, Arantxa Pérez-García, Alberto Benguria, Carlos Torroja, Virginia G. de Yébenes, Almudena R. Ramiro

**Affiliations:** 1 B Cell Biology Lab, Centro Nacional de Investigaciones Cardiovasculares, Madrid, Spain; 2 Genomics Unit, Centro Nacional de Investigaciones Cardiovasculares, Madrid, Spain; 3 Bioinformatics Unit, Centro Nacional de Investigaciones Cardiovasculares, Madrid, Spain

## Abstract

Activation-induced deaminase (AID) initiates antibody diversification in germinal center (GC) B cells through the deamination of cytosines on immunoglobulin genes. AID can also target other regions in the genome, triggering mutations or chromosome translocations, with major implications for oncogenic transformation. However, understanding the specificity of AID has proved extremely challenging. We have sequenced at very high depth >1,500 genomic regions from GC B cells and identified 275 genes targeted by AID, including 30 of the previously known 35 AID targets. We have also identified the most highly mutated hotspot for AID activity described to date. Furthermore, integrative analysis of the molecular features of mutated genes coupled to machine learning has produced a powerful predictive tool for AID targets. We also have found that base excision repair and mismatch repair back up each other to faithfully repair AID-induced lesions. Finally, our data establish a novel link between AID mutagenic activity and lymphomagenesis.

## Abstract (precis)

Álvarez-Prado et al. report a detailed map of AID-induced off-target mutations and identify molecular features that predict gene mutability. They identify a novel AID hotspot and demonstrate that base excision and mismatch repair back up each other to repair most AID deamination events.

## Introduction

Activation-induced deaminase (AID) is a crucial enzyme for the immune response because it generates high-affinity and switched antibodies in germinal center (GC) B cells by somatic hypermutation (SHM) and class switch recombination (CSR; Muramatsu et al., 2000; Revy et al., 2000). AID initiates SHM and CSR through the deamination of deoxycytidine residues into deoxyuridines on the DNA of Ig genes (Muramatsu et al., 2000; Petersen-Mahrt et al., 2002; Di Noia and Neuberger, 2007; Stavnezer et al., 2008). The resulting U:G mismatch can be alternatively recognized and processed by base excision repair (BER) or mismatch repair (MMR) pathways, leading either to point mutations, in the case of SHM, or to double-strand breaks (DSBs) followed by a recombination reaction, in the case of CSR (Di Noia and Neuberger, 2007; Stavnezer et al., 2008; Reynaud et al., 2009; Methot and Di Noia, 2017). Although AID activity has a strong preference for Ig genes, it can also target other genes, giving rise to point mutations (Shen et al., 1998; Pasqualucci et al., 2001; Liu et al., 2008) or oncogenic chromosome translocations (TCs; Ramiro et al., 2004, 2006; Robbiani et al., 2008). Understanding AID specificity, or targeting, has been hindered by the technical challenge of detecting AID-induced mutations, which occur at very low frequencies. Here, we have used next generation sequencing to directly measure raw AID mutational activity on a broad representation of the genome and thus gather conclusions on AID specificity, DNA repair, and lymphomagenesis.

## Results and discussion

### Capture-based deep sequencing allows high-throughput identification of AID targets

**Figure 1.**
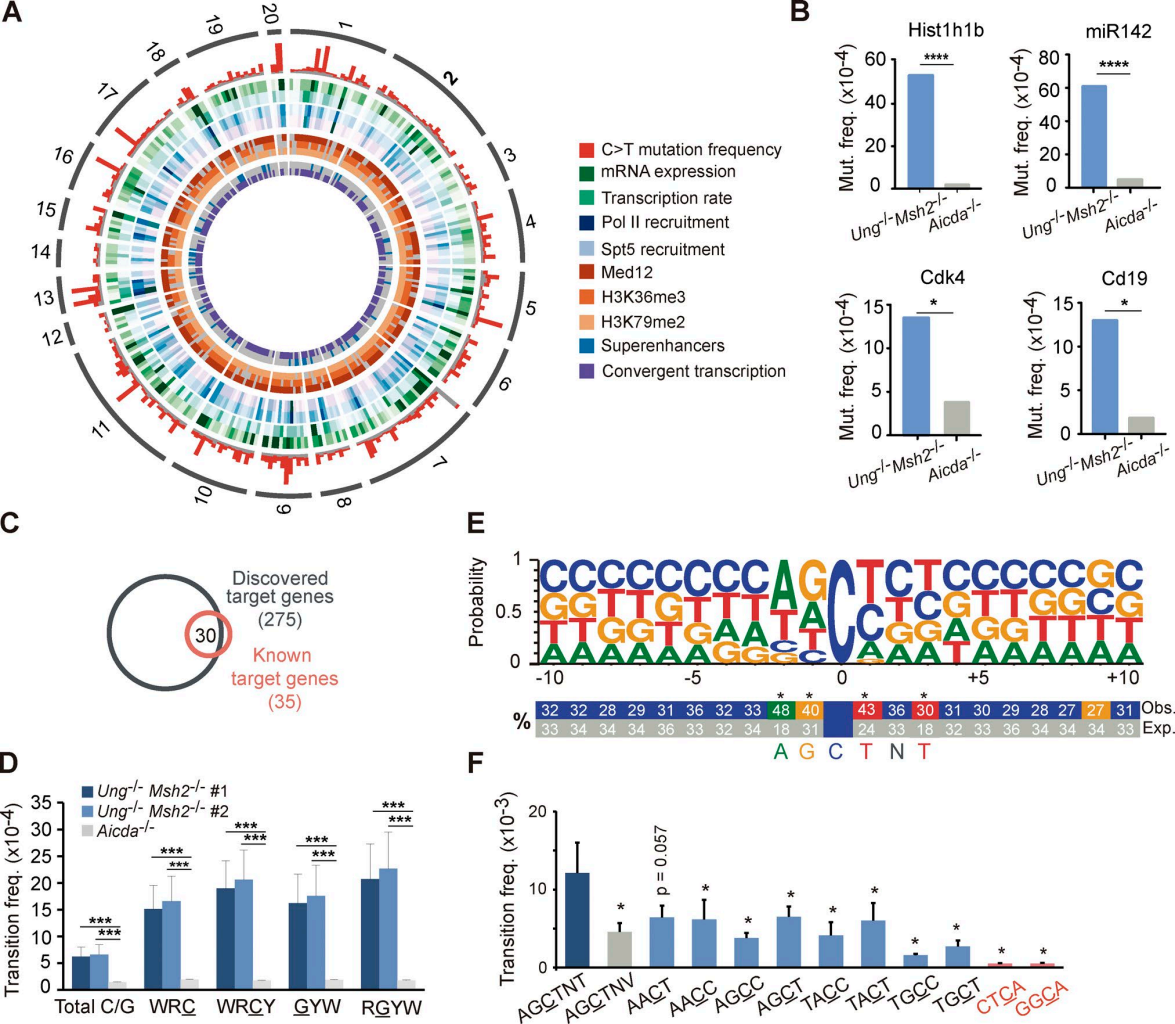
**High-throughput analysis of AID-induced mutations.** DNA from Peyer's patch GC B cells was captured with a probe library for 1,588 genomic regions (Table S1) and deep sequenced. AID targets were identified as those regions accumulating significantly more C→T transition mutations in *Ung^−/−^Msh2^−/−^* than in *Aicda^−/−^* mice (Table S2; FDR ≤0.05, one-tail Fisher test and Benjamini-Hochberg correction; two independent experiments; see Materials and methods). **(A)** Circos plot representation of the AID targets identified in this study and their associated molecular features. The outer ring shows chromosome location and is followed by C→T transition mutation frequency in *Ung^−/−^Msh2^−/−^* (red) and *Aicda^−/−^* (gray) mice. **(B)** Validation of representative AID targets by Sanger sequencing (one-tail Fisher test; Table S3). **(C)** Overlap between the targets discovered in this study and previously reported AID targets. **(D)** Mean transition frequency in total C/G nucleotides and in C/G within WRC(Y)/(R)GYW hotspots (W = A/T; R = G/A; Y = C/T) of the 291 AID targets (two-tailed Student’s *t* test; two independent experiments). **(E)** Logo representation of the sequence context of mutated cytosines (mutation frequency ≥4 × 10^−3^). Statistically significant enrichment of nucleotides surrounding the mutated C is indicated (*, FDR ≤10^−3^, one-tail Fisher test and Bonferroni correction; see Materials and methods), and numbers indicate percentages. **(F)** Mean mutation frequency of cytosines within the indicated motifs (dark blue bar, newly identified hotspot; gray bar, control motif for newly identified hotspot; light blue bars, WRCY hotspots; red bars, random four-nucleotide motifs; two-tailed Mann-Whitney test). *, P ≤ 0.05; ***, P < 10^−3^; ****, P < 10^−4^. Error bars depict SEM.

To explore the scope of AID-induced mutations at a high-throughput scale, we designed a capture library against 1,588 regions corresponding to 1,379 different genes as a representation of the B cell genome (Table S1; see Design of DNA capture library in the Materials and methods). Genomic DNA from GC B cells was isolated, captured, and deep sequenced (Fig. S1, A and B). We made use of a mouse model deficient for both BER and MMR pathways (*Ung^−/−^Msh2^−/−^* mice). In the absence of BER and MMR, AID-induced U:G mismatches remained unprocessed and were replicated over, thus leaving behind almost solely C→T and G→A transitions, the footprint of AID deamination events on DNA (Rada et al., 2004; Methot and Di Noia, 2017). This approach allowed an extremely efficient enrichment and sequencing depth (Fig. S1, A and B). We found a set of 291 genomic regions (corresponding to 275 different genes) that were reproducibly mutated in *Ung^−/−^Msh2^−/−^* GC B cells when compared with *Aicda*^−/−^ GC B cells (q ≤ 0.05; Fig. 1 A; Fig. S1, C–E; and Table S2; representative targets were validated by Sanger sequencing; Fig. 1 B and Table S3). Importantly, the 275-gene target collection included 30 of the 35 previously known AID targets, such as *Bcl6*, *Pim1*, *RhoH*, *Pax5*, and *Cd83* (Fig. 1 C and Table S2; Pasqualucci et al., 2001; Liu et al., 2008; Methot and Di Noia, 2017). Mutations detected in the 291-target regions strongly accumulated in AID mutational hotspots (WRC(Y)/(R)GYW; underlined letters specify deaminated nucleotides; W = A/T; R = A/G; Y = C/T; Fig. 1 D; Rogozin and Kolchanov, 1992). Finally, we found that our 275-target set included a big proportion of genes subject to DSBs or chromosome TCs (Fig. S1 F; Chiarle et al., 2011; Klein et al., 2011; Staszewski et al., 2011; Qian et al., 2014; Dong et al., 2015). Thus, our deep sequencing approach has allowed the discovery of an unprecedented, massive collection of AID targets.

### Identification of AGCTNT as a novel AID hotspot

To gain insights into the local sequence preference of AID, we first analyzed the mean mutation frequency at individual WRCY/RGYW hotspots across all 291 AID targets and found a wide range of mutability, with AACT and AGCT as the top mutated hotspots in both strands of DNA, which may reflect an intrinsic preference for AID deaminase activity. Next, we performed an unbiased analysis of the sequence context of mutated cytosines. We found that A, G, and T nucleotides were the preferred nucleotides at −2, −1, and +1 positions (Pérez-Durán et al., 2012; Wei et al., 2015; Yeap et al., 2015), respectively, but we further uncovered a significant preference for T at +3 (Fig. 1 E and Fig. S2). Indeed, cytosines lying at the AGCTNT motif were significantly more mutated than those in AGCTNV (where V is A, C, or G) or than other WRCY/RGYW hotspots (Fig. 1 F and Fig. S2, A and B). Thus, our study has revealed AGCTNT as a novel and the most highly mutated AID hotspot identified so far.

### Prediction of AID targets

**Figure 2.**
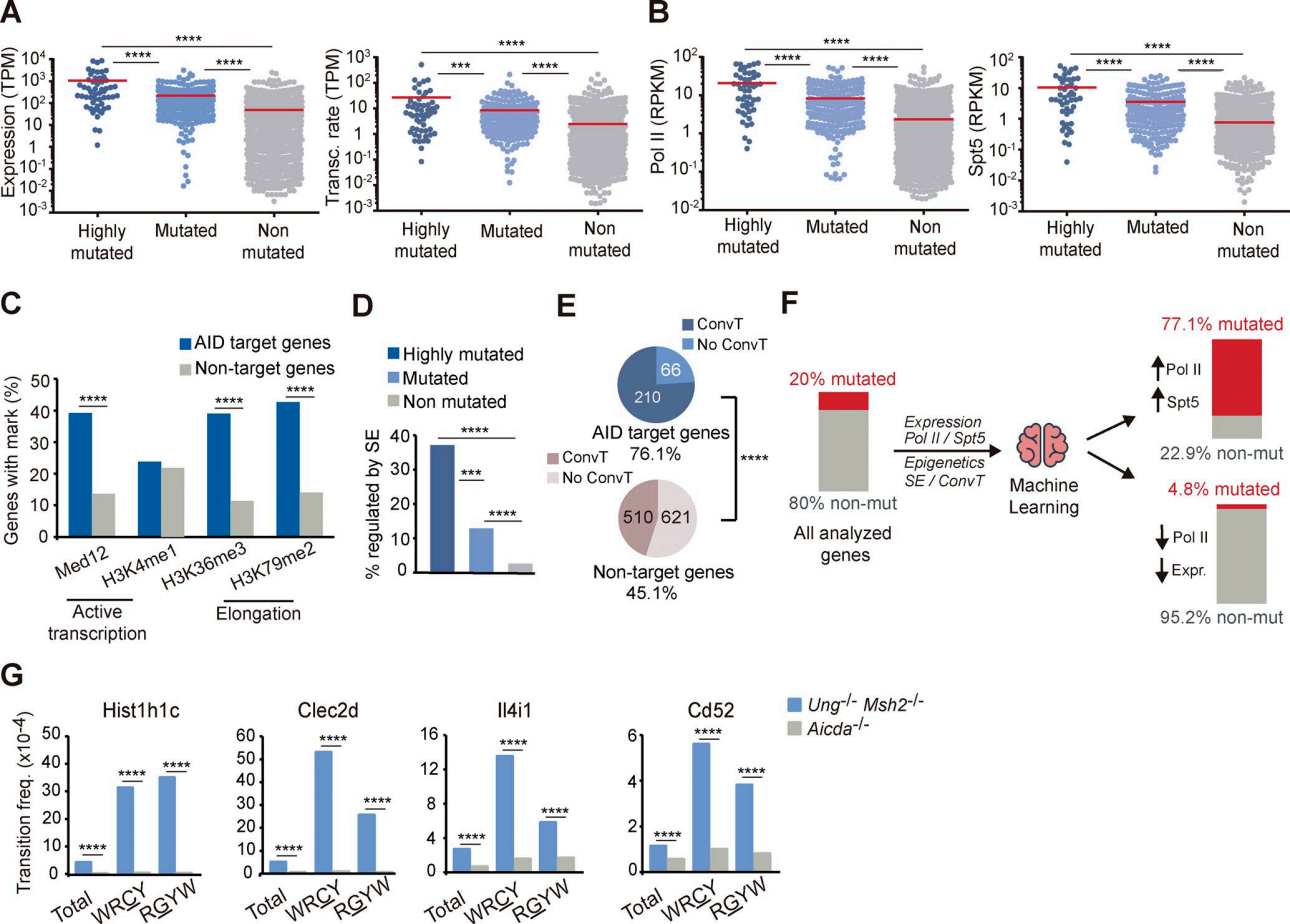
**Molecular features of AID targets predict mutability. (A)** Expression level of highly mutated (top 20% mutated genes, C→T transition frequency >3 × 10^−4^), mutated (rest of mutated), and nonmutated genes in Peyer's patch GC B cells as measured by RNA-Seq and transcription rate of AID targets in GC B cells from lymph nodes as measured by GRO-Seq. TPM, transcripts per million. **(B)** Recruitment of RNAPolII and Spt5 to AID targets and nontargets measured in in vitro activated splenic B cells by ChIP-Seq. RPKM, reads per kilobase per million reads mapped. **(C)** Transcription and transcription elongation marks in AID targets and nontargets by ChIP-Seq analysis of in vitro activated splenic B cells (Med12, H3K4me1, H3K36me3, and H3K79me2). **(D)** Proportion of highly mutated, mutated, and nonmutated genes regulated by superenhancers (SE) in GC B cells (see Materials and methods). **(E)** GRO-Seq analysis of convergent transcription (ConvT) in AID targets and nontargets from GC splenic B cells obtained from SRBC-immunized mice. **(F)** Representation of the machine-learning approach used for AID target prediction. **(G)** Validation of representative genes predicted to be mutated by the model by PCR-Seq. Statistical tests: two-tailed Student’s *t* test (A, B, and G) and one-tailed Fisher test (C–E). ***, P < 10^−3^; ****, P < 10^−4^.

Using the uniquely large set of AID-mutated genes identified in this study, we performed a comprehensive analysis of molecular features that associate with SHM, including transcription, epigenetic marks, and regulatory sequences (Fig. 1 A; Storb, 2014; Methot and Di Noia, 2017). We first observed that transcription levels and transcription rates are significantly higher in AID targets than in nontargets and that this difference is even higher for highly mutated targets (Fig. 2 A). We also found that RNAPolII and the stalling factor Spt5, previously described to associate with AID (Nambu et al., 2003; Pavri et al., 2010), show higher binding density within AID mutational targets (Fig. 2 B). Likewise, AID targets were enriched in marks of active enhancers and transcriptional elongation, such as Med12, H3K36me3, and H3K79me2 (Fig. 2 C). Finally, we found that primary AID targeting, as measured by AID mutations in the absence of repair, also focuses preferentially in the vicinity of superenhancers (Fig. 2 D) and in regions subject to convergent transcription (Fig. 2 E; Meng et al., 2014; Qian et al., 2014). Together, our mutagenesis study shows that several mechanisms linked to transcription are critical for AID activity, as suggested in previous studies (Nambu et al., 2003; Pavri et al., 2010; Meng et al., 2014; Qian et al., 2014; Wang et al., 2014). Our data also indicate that AID targeting cannot be defined by any of these features alone. To approach whether a combination of these molecular features could be used to predict AID targeting, we developed a prediction model using a machine-learning algorithm, fed with the collection of genes analyzed here together with the set of molecular features described in Fig. 2 (A–E) (Fig. S3, A and B; see Machine learning to predict AID targets in the Materials and methods for details). We found that a combination of high-density RNAPolII and Spt5 binding, found in 2.3% of genes in the whole genome (Fig. S3 B), predicts AID specificity with 77% probability (P < 0.001; Fig. 2 F and Fig. S3 A). Conversely, low RNAPolII binding combined with low gene expression predicted the absence of mutations for 95% of genes (Fig. 2 F). To test the accuracy of our prediction model, we analyzed the mutation frequency of a new collection of genes (not included in our capture library) with high-density RNAPolII and Spt5 binding (Fig. S3 C and Table S4). We found that 11/12 of the analyzed genes were significantly mutated (Table S4 and Fig. 2 G). Indeed, two genes (*Hist1h1c* and *Clec2d*) were mutated at the range of the top 20% mutated genes at frequencies similar to those found in *Pax5* or *Rhoh* (Table S2 and Table S4). Thus, we have built a powerful predictive tool for AID activity.

### BER and MMR back up each other to faithfully repair AID-induced lesions

**Figure 3.**
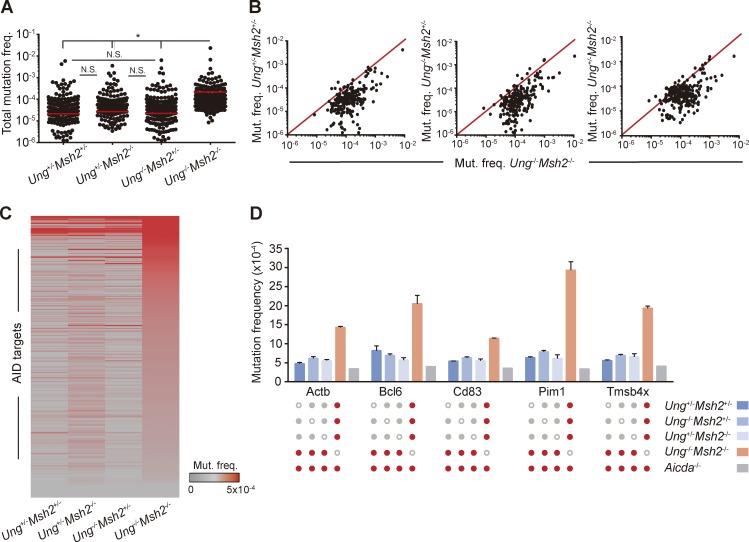
**BER and MMR back up each other to error-free repair AID-induced lesions. (A and B)** Total mutation frequency of AID targets in *Ung^+/−^Msh2^+/^*^−^, *Ung^−/−^Msh2^+/−^*, and *Ung^+/−^Msh2^−/−^* GC B cell mice compared with that of *Ung^−/−^Msh2^−/−^* mice (mean of two independent experiments; see Materials and methods; Table S2). **(C)** Heat map representation of AID targets in *Ung^+/−^Msh2^+/^*^−^, *Ung^−/−^Msh2^+/−^*, *Ung^+/−^Msh2^−/−^*, and *Ung^−/−^Msh2^−/−^* GC B cells. **(D)** Mutation frequency of representative genes in *Ung^+/−^Msh2^+/−^, Ung^−/−^Msh2^+/−^, Ung^+/−^Msh2^−/−^*, *Ung^−/−^Msh2^−/−^*, and *Aicda^−/−^* GC B cells. Red dots indicate statistically different mutation frequencies between the indicated genotypes. Mutation frequency found in *Aicda^−/−^* mice was substracted before plotting A–C. (A and D) Two-tailed Student’s *t* test; *, P ≤ 0.05. Error bars depict SEM. N.S., not significant.

BER and MMR act downstream of AID-induced U:G mismatches so that UNG is critical for the generation of transversions at C:G pairs while MSH2 facilitates the introduction of mutations at A:T pairs (Frey et al., 1998; Phung et al., 1998; Rada et al., 1998, 2002, 2004; Methot and Di Noia, 2017). UNG and MSH2 can also promote conventional, faithful repair of AID-induced U:G mismatches (Liu et al., 2008; Pérez-Durán et al., 2012). To explore the contribution of BER and MMR to AID mutagenic activity, we analyzed GC B cells from single-deficient *Ung^+/−^Msh2^−/−^* and *Ung^−/−^Msh2^+/−^* mice and from control *Ung^+/−^Msh2^+/−^* mice and compared the mutation frequency of the 291 AID target regions identified in this study (Table S2). We found similar mean mutation frequencies in B cells deficient for UNG alone, MSH2 alone, or proficient for both, whereas AID targets harbored significantly more mutations in the combined absence of UNG and MSH2 (Fig. 3, A and B). Indeed, only a small proportion (∼6%) of the genes mutated in *Ung^−/−^Msh2^−/−^* cells was also mutated in single-knockout and double-heterozygous cells (Fig. 3 C and Table S2). Moreover, we found that classical AID off targets, such as *Bcl6* or *Pim1*, although mutated in all genotypes analyzed, harbored a significantly bigger load of mutations in *Ung^−/−^Msh2^−/−^* cells than in *Ung^+/−^Msh2^−/−^*, *Ung^−/−^Msh2^+/−^*, or *Ung^+/−^Msh2^+/−^* cells (Fig. 3 D). Together, these data indicate that BER and MMR back up each other to faithfully repair most of the AID-induced lesions in GC B cells.

### AID targets are recurrently mutated in human lymphomas

**Figure 4.**
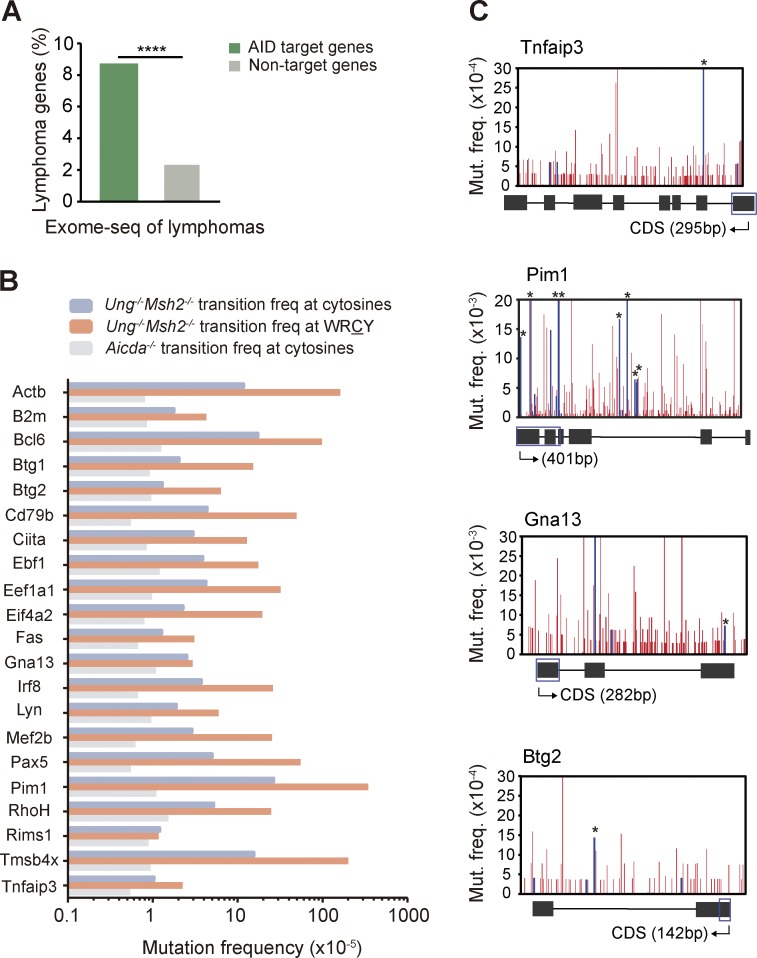
**AID targets are recurrently mutated in human lymphomas. (A)** AID targets are enriched in genes involved in lymphoma development. Percentage of lymphoma genes within AID target and nontarget genes. Annotation was done from public data on human lymphoma sequencing (see Materials and methods; two-tailed Fisher test; ****, P < 10^−4^). **(B)** Mutation frequency in total C/G nucleotides and C/G nucleotides within WRC(Y)/(R)GYW hotspots (W = A/T; R = G/A; Y = C/T) of the 21 AID target genes involved in human DLBCL development analyzed in *Ung^−/−^Msh2^−/−^* mice (mean of two independent experiments; see Materials and methods). **(C)** Mutation profiles of representative DLBCL genes analyzed in *Ung^−/−^ Msh2^−/−^* mice. Blue bars indicate mutations identical to those found in human lymphoma tumor samples (Table S5); asterisks indicate mutations occurring in a WRC(Y) hotspot. The diagrams below the graphs represent the complete gene (not to scale), and blue boxes indicate the region depicted above. Mutation frequency found in each nucleotide in *Aicda^−/−^* mice was subtracted before plotting.

We next assessed the contribution of AID off-target mutations to B cell–derived malignancies by making use of available sequencing data on human lymphomas. We found that AID targets are significantly enriched in genes mutated in human B cell lymphomas (see Annotation of AID targets in the Materials and methods for details; Fig. 4 A). Indeed, 21/275 (7.6%) of our set of AID target genes are mutated in diffuse large B cell lymphomas (DLBCLs; Fig. 4 B), a highly prevalent, aggressive form of lymphoma (Shaffer et al., 2012). Lymphoma genes mutated by AID included *Bcl6*, *RhoH*, *Pim1*, *Ebf1*, *Eif4a2*, and *Pax5*, which is in agreement with previous studies (Shen et al., 1998; Pasqualucci et al., 2001; Liu et al., 2008). In addition, we identified nine novel genes mutated in human DLBCLs that accumulate AID-induced mutations (Fig. 4 B), including *Mef2b*, *Lyn*, *Tnfaip3*, *Gna13*, and *Irf8*. Remarkably, we found many instances where the exact same mutations described in human lymphoma genes were also found in the AID targets identified in this study in nontransformed mouse B cells (Fig. 4 C and Table S5). Together, these results suggest that off-target AID mutagenic activity can contribute to GC-associated lymphomagenesis.

Until now, the study of AID specificity has been hindered by the technical challenge of detecting AID-induced mutations; indeed, only a limited number of genes has been directly interrogated for AID-mediated mutagenesis (Pasqualucci et al., 2001; Liu et al., 2008; Methot and Di Noia, 2017). However, genome-wide AID specificity has been inferred from high-throughput analysis of AID binding, which does not warrant AID activity, AID-induced DSBs, or chromosomal TCs, which involve complex processing of the initial lesion induced by AID (Chiarle et al., 2011; Klein et al., 2011; Staszewski et al., 2011; Yamane et al., 2011; Meng et al., 2014; Qian et al., 2014). The strategy developed in this study has provided an unprecedented scope to the analysis of AID targeting: we describe here the broadest collection of AID mutational targets (275 genes) to date, 10-fold larger than the previously known targets. The strength of this analysis is well supported by the confirmation of the vast majority of previously identified AID targets and the validation of targets by conventional Sanger sequencing.

Here, we have integrated our mutation data with a collection of molecular features of GC B cells to feed a machine-learning algorithm. According to the machine-learning tree generated here, the combined binding of Spt5 and RNAPolII at high density is the best predictor for AID mutability, although additional combinations of transcriptional traits bear some predictive power as well. Furthermore, we have performed independent experimental validation showing that randomly picked Spt5^high^RNAPolII^high^ genes indeed are very frequently mutated by AID. This is, to our knowledge, the first instance of a tool that successfully predicts the potential of a gene to be targeted by AID. Regarding the fate of AID-induced lesions, BER and MMR have long been known to broaden the diversity of SHM with an apparent perverted recruitment of error-prone polymerases and to do so in a cooperative manner (Rada et al., 2004; Di Noia and Neuberger, 2007; Methot and Di Noia, 2017). The mechanisms responsible for the error-free versus error-prone activity of UNG and MSH2 are far from understood, and both gene-specific and local sequence contexts may play a role in defining the fate of the U:G resolution (Liu et al., 2008; Pérez-Durán et al., 2012; Wei et al., 2015). Strikingly, here we show that the fate of the majority of off-target lesions induced by AID is to undergo faithful repair by BER and MMR and that, again, both pathways can back up each other in this task with only a minor fraction of the mutations escaping them. Whether this reflects gene-specific qualities or is the consequence of excessive mutation load will deserve further investigation. We would speculate that a minor fraction of unrepaired mutations in prolymphomagenic genes could provide cell growth advantage and account for the predominance of AID-mediated mutations in lymphomas. Regardless of oncogenic relevance, it is remarkable that even though our mutation analysis was performed in nontransformed cells, we could detect individual AID-induced mutations that are recurrently mutated in lymphoma. Thus, our results yield a novel perspective on the contribution of AID activity to B cell transformation through the introduction of mutations.

We expect our mutational study will be valuable for other research questions, including validation of novel molecular mechanisms involved in AID targeting, prediction of novel targets, or assessment of cancer-associated mutations. Furthermore, similar approaches would be of immediate interest to broaden our knowledge on the role of AID or other mutagenic activities not only in B cell lymphomas, but also in malignancies from any origin.

## Materials and methods

### Mice

*Ung* and *Msh2* mutant mice used in this study were generated by crossing *Ung^−^*^/^*^−^* mice (Nilsen et al., 2000) and *Msh2*^−/−^ mice (Reitmair et al., 1995). *Aicda*^−/−^ mice have been previously described (Muramatsu et al., 2000). Mice were housed in specific pathogen-free conditions. Male and female mice between 20 and 28 wk were used for the experiments. The number of animals per group to detect biologically significant effect sizes was calculated using an appropriate statistical sample size formula. All experiments were done in concordance with EU Directive 2010/63EU and Recommendation 2007/526/EC regarding the protection of animals used for experimental and other scientific purposes, enforced in Spanish law under RD 53/2013.

### Design of DNA capture library

A set of 1,379 mouse genes was selected as a representation of the genome (Table S1). 85% of all genes were randomly picked, ensuring even representation of chromosomal location by bioinformatic analysis and unbiased biological function. ∼15% of the library corresponded to previously known AID targets (Müschen et al., 2000; Pasqualucci et al., 2001; Gordon et al., 2003; Liu et al., 2008; Robbiani et al., 2009; Pavri et al., 2010), IgH probes, and other controls. Probes were designed in eArray (Agilent) to capture the first 500 bp downstream of each transcriptional start site (TSS) of each of the 1,379 genes. Because various genes contained more than one predicted TSS, the library includes a total of 1,588 different genomic regions. Library design included 50 extra nucleotides at both ends of each region to optimize the capture yield. A custom target enrichment capture library was then synthesized by the manufacturer (SureSelectXT; Agilent).

### DNA capture and sequencing

GC (*Cd19^+^Fas^+^GL7^+^*) B cells were isolated from Peyer’s patches of *Ung^+/−^Msh2^+/−^* (*n*_1_ = 10; *n*_2_ = 11), *Ung^−/−^Msh2^+/−^* (*n*_1_ = 46; *n*_2_ = 8), *Ung^+/−^Msh2^−/−^* (*n*_1_ = 46; *n*_2_ = 2), and *Ung^−/−^Msh2^−/−^* (*n*_1_ = 37; *n*_2_ = 8) littermates and *Aicda^−/−^* (*n* = 31 mice) mice by sorting in a FACSAria cell sorter (BD Biosciences) after staining with anti–mouse antibodies to *Cd19*, *Fas*, and *GL7* (BD Biosciences). Genomic DNA was isolated by standard procedures and quantified in a fluorometer (Qubit; Invitrogen). DNA capture, library preparation, and DNA sequencing were performed by the Genomics Unit at Centro Nacional de Investigaciones Cardiovasculares (CNIC). In brief, DNA was fragmented in a sonicator (Covaris) to ∼200 nucleotide-long (mean size) fragments and purified using AMPure XP beads (Agencourt). Quality was assessed with the 2100 Bioanalyzer (Agilent). Then, fragment ends were repaired, adapters were ligated, and the resulting library was amplified and hybridized with our custom SureSelectXT library of RNA probes. DNA–RNA hybrids were then captured by magnetic bead selection. After indexing, libraries were single-end sequenced in a HiSeq 2500 platform (Illumina).

### Target enrichment assessment by quantitative RT-PCR

*Noxa1*, *Ostn*, and *Pcna* amplifications were quantified with green assay (SYBR; Applied Biosystems) in a real-time PCR system (AB7900 Standard; AbiPrism). *Gapdh* amplifications were used as normalization controls. The following primers were used: *Gapdh* (forward), 5′-TGAAGCAGGCATCTGAGGG-3′; *Gapdh* (reverse), 5′-CGAAGGTGGAAAGTGGGAG-3′; *Ostn* (forward), 5′-CATAGTGTTGCTGTGGTT-3′; *Ostn* (reverse), 5′-CATTATATTGGTCTGCTGTT-3′; *Noxa1* (forward), 5′-CGCGGGACAGCAATGAGAAG-3′; *Noxa1* (reverse), 5′-CCATCTACTCAGTTTCAAGGA-3′; *Pcna* (forward), 5′-CTCCAGCACCTTCTTCAG-3′; and *Pcna* (reverse), 5′-TCTCATCTAGTCGCCACA-3′.

SDS software (Applied Biosystems) was used for analysis of the data.

### Sanger sequencing

Regions to be sequenced were amplified from 160–200-ng genomic DNA in four independent reactions to minimize possible PCR biases. The following primers were used: *Hist1h1b* (forward), 5′-ATGCCTTAGACTTCACCGCC-3′; *Hist1h1b* (reverse), 5′-TTGTAACCTTGAGTCGCCGC-3′; *miR142* (forward), 5′-CGGTCCCTGGGAAGTTACAC-3′; *miR142* (reverse), 5′-AACGAGAGGCAAACAGTCTTCA-3′; *Cd19* (forward), 5′-GCCCCTCTTCCCTCCTCATA-3′; *Cd19* (reverse), 5′-CCTGCACCCACTCATCTGAA-3′; *Cdk4* (forward), 5′-TCTGGCAGCTGGTCACATGG-3′; and *Cdk4* (reverse), 5′-GATCACCAGCTAGTCGTCCC-3′. Amplification reactions were carried in a final volume of 25 µl using 2.5 U Pfu Ultra HF DNA polymerase (Agilent) and the following PCR setup: 95°C for 2 min, 25 (*Cd19* and *Cdk4*) or 26 cycles (*miR142* and *Hist1h1b*) of denaturation at 94°C for 30 s, annealing at 57°C (*miR142* and *Hist1h1b*) or 58°C (*Cd19* and *Cdk4*) for 30 s, extension at 72°C for 1 min, and a final stage of 72°C for 10 min. PCR products were purified from a 1% agarose gel (Illustra Gel Band Purification kit; GE Healthcare) and cloned into pGEMT vector (Promega). Competent DH5α *Escherichia coli* bacteria were transformed with the constructs, and individual colonies (192–288 per gene) were grown in 96-well plates. Plasmidic DNA was then isolated (Plasmid MiniPrep kit; Millipore) and sequenced by Sanger sequencing using SP6 universal primer. Sequence analysis was performed using SeqMan software (Lasergene).

### PCR-Seq to validate the machine-learning approach

40–50 ng of genomic DNA was amplified using the following primers: *Apobec3* (forward), 5′-GTCTTCCATAGCCTGCTCACA-3′; *Apobec3* (reverse), 5′-TAGCTGACTGGTGTGGTTCC-3′; *Aurkaip1* (forward), 5′-ACTTGTCACTTCCGCAGTCC-3′; *Aurkaip1* (reverse), 5′-CCATCCCCAAGTCAGGTGTG-3′; *Ccdc17* (forward), 5′-TCTTTTCTGTCCAGTCCGCC-3′; *Ccdc17* (reverse), 5′-ACAAATGGGCAGAGTCAGGG-3′; *Cd52* (forward), 5′-TACTGCCGCACACATGACTC-3′; *Cd52* (reverse), 5′-TGAGGTGGGAAGCCAAACAT-3′; *Cd68* (forward), 5′-AGGGGCTGGTAGGTTGATTG-3′; *Cd68* (reverse), 5′-GGAGTCAGGACTGGATTTGAC-3′; *Cd69* (forward), 5′-TCTAAAGGTTTTGAGACCCCC-3′; *Cd69* (reverse), 5′-TGAAGCCTCATCAACGCACT-3′; *Clec2d* (forward), 5′-GGCTCCTGACCTTGAAATGC-3′; *Clec2d* (reverse), 5′-AGGCAACTTCTGCCACTATGC-3′; *Coro1a* (forward), 5′-AGGGCTCTGGGGTTCTACTT-3′; *Coro1a* (reverse), 5′-GGAAATGACCACGGGGGTTT-3′; *Hist1h1c* (forward), 5′-CTCTATCGGCGTACTGCCAC-3′; *Hist1h1c* (reverse), 5′-ATCGAGTCCCTTGCAACCTT-3′; *Il4i* (forward), 5′-ATTCCCGAGGGAGGTGAGTG-3′; *Il4i* (reverse), 5′-GGTAGCTTCTCTCCGTCACAC-3′; *Maz* (forward), 5′-GTCAACAAAGAACCCCTCCCT-3′; *Maz* (reverse), 5′-CACCTGTCCCCTGAGTTGTG-3′; *Trex1* (forward), 5′-GCCTAACAGGTTTGATTGTCC T-3′; and *Trex1* (reverse), 5′-TAGGCTGAGCACTCCCAGTC-3′. Amplification reactions were carried in a final volume of 25 µl using 2.5 U Pfu Ultra HF DNA polymerase (Agilent; 95°C for 2 min, 26 cycles of 94°C for 30 s, 55°C for 30 s, 72°C for 1 min, and a final stage of 72°C for 10 min). PCR products were purified and fragmented using a sonicator (Covaris), and libraries were prepared by the CNIC Genomics Unit according to the manufacturer’s instructions (NEBNext Ultra DNA Library Prep; New England Biolabs). Sequencing was performed in a HiSeq 2500 platform (Illumina). Analysis was performed as previously described (Pérez-Durán et al., 2012).

### Gene expression profiling by RNA-Seq

GC (*CD19^+^Fas^+^GL7^+^*) and resting (*CD19^+^Fas^−^GL7^−^*) B cells were sorted from Peyer’s patches of littermate 12-wk-old WT C57BL/6 mice. Three biological replicates were analyzed, each composed of a pool of five female mice. RNA was purified from pellets of 2–2.5 × 10^4^ cells, and DNaseI treatment was applied to avoid DNA contamination (RNAeasy MiniKit; Qiagen). RNA quality was assessed with the 2100 Bioanalyzer, showing high RNA purity and integrity. Sequencing libraries were prepared by the CNIC Genomic Unit according to the manufacturer’s protocol (NEB NEXT Ultra RNaseq Library Prep kit; New England Biolabs) from 100 ng RNA per replicate and sequenced in a HiSeq 2500 platform.

### Computational analysis

#### Pipeline to identify and annotate AID-induced mutations

Raw reads were demultiplexed by Casava v1.8 to generate a fastq file that was aligned to the mouse genome (NCBIm37 v61 Feb 2011) with Novoalign 2.08.01 (command line options: -o SAM -F ILM1.8 -H -r None -q 2). Sam files were processed with Samtools 0.1.19 to generate a sorted bam file that was piped to a custom Perl script for the analysis of AID mutations. In brief, the software analyzes the regions of interest in the bam file, annotates hotspots, localizes and suppresses annotated single nucleotide polymorphism positions (Sanger Mouse Genomes Project SNP and Indel Release v2), and reports relevant information about AID activity. AID targets were identified as those genes accumulating significantly more C→T transition mutations in *Ung^−/−^Msh2^−/−^* than in *Aicda^−/−^* mice (false discovery rate [FDR] ≤0.05, one-tail Fisher test and Benjamini-Hochberg correction).

Mutation frequencies were calculated as follows:

$$\mathrm{Total mutation freq}=\frac{\mathrm{Total number of mutations}}{\mathrm{Total sequenced length}},$$

Mutation freq C/G=(Mutated cytosines+Mutated guanines)(Seq length cytosines+Seq length guanines),

and

Mutation freq WRC(Y)/(R)GYW =(Mutated cytosinesWRC(Y)+Mutated guanines(R)GYW)(Seq length cytosinesWRC(Y)+Seq length guanines(R)GYW).

(Only cytosines in WRC(Y) and guanines in (R)GYW were considered to calculate mutation frequency at hotspots.)

#### Integration of AID targets with public data on TC and DSB occurrence

The bar graph included in Fig. S1 F represents overlaps in the 1,375 genes analyzed in this study (divided into mutated and nonmutated genes) and genes where TCs or DSBs occur in B cells: Meng et al. (2014) refer to TC sites identified by HTGTS in αCD40+IL4-activated B cells as published in Table S2 from their study; Klein et al. (2011) refer to TC sites identified by TC-Seq in IgH^I-Sce^ LPS+IL4-activated B cells as published in Table S4 from their study; Chiarle et al. (2011) refer to TC sites identified by HTGTS in c-myc^25xI-SceI^ αCD40+IL4-activated B cells as published in Table S3 (significant hits at P ≤ 0.05) from their study; Qian et al. (2014) refer to DSBs identified by replication protein A (RPA) differential recruitment (RPA–chromatin immunoprecipitation [ChIP]) in Ig*k*AID 53BP1^−/−^ in vitro activated B cells as published in Table S1 A from their study; and Staszewski et al. (2011) refer to DSBs identified by Nbs1 binding (ChIP-on-ChIP) in LPS+αIgD-dextran+BLySS-activated B cells as published in Table S1 (P ≤ 0.05) from their study.

#### Sequence context of mutated cytosines

The sequence context of mutated cytosines (C→T transition frequency ≥4 × 10^−3^) was analyzed in a window of 10 nucleotides. Logo representation was done using WebLogo3, and the percentage of each nucleotide in each position surrounding the mutated cytosine was calculated by a custom Perl script. Enrichment for adenosine, guanine, cytosine, or thymine was tested against the sequence context of all cytosines present in the 1,588 regions analyzed in this study (one-tailed Student’s *t* test + Bonferroni correction).

#### Gene expression profiling by RNA-Seq

After demultiplexing by Casava v1.8, read quality was assessed by FASTQC, and sequencing adapters were removed from sequence reads by cutadapt v1.9. The resulting reads were aligned to and quantified on the mouse transcriptome (NCBIm38 v75, Feb 2014) using RSEM v1.2.25 with the following parameters: -p 3–time–output-genome-bam–sampling-for-bam–bowtie-e 60–bowtie-m 30–bowtie-chunkmbs 512–fragment-length-mean 180–fragment-length-sd 50.

#### Transcription rate analysis (GRO-Seq)

Reads were mapped to the mouse genome (mm9/NCBI37) using bowtie2, and uniquely mapped, nonredundant reads were kept. Reads mapping in ±1 kb from TSSs were quantified and summarized at the gene level using HTSeq.

#### PolII and Spt5 recruitment

Quantification of PolII and Spt5 recruitment was extracted from Table S3 A in Pavri et al. (2010).

#### Superenhancer analysis

Data were extracted from the catalog of superenhancers that overlap with gene bodies identified in GC B cells as published in Table S3 in Meng et al. (2014) (GEO accession no. GSE62296).

#### Epigenetic mark analysis

Sequencing data (fastq files) for each epigenetic mark were aligned to the mouse genome (NCBIm37 v61, Feb 2011) using bowtie 1.1.1 (command line options:–best -m1 -n2 -p2). Alignment files were processed by Samtools 0.1.19 to generate a sorted bam file. Peak calling was done using MACS2 (v2.1.0.20140616) according to the optimal parameters for a histone modification status profiling as reported by the creators of the tool (Feng et al., 2011). Mapping of annotated peaks to genes was done using GREAT (version 3.0.0).

#### Convergent transcription analysis (GRO-Seq)

Convergent transcription data analysis was performed as described in Meng et al. (2014). In brief, reads were mapped to the mouse genome (mm9/NCBI37) using bowtie2, and uniquely mapped, nonredundant reads were kept. HOMER (v4.6) was used with default parameters to identify transcribed regions from both strands and bedtools (v2.24) to find and annotate ConvT regions (regions where >100 bp of sense and antisense transcription overlap occurs).

#### Machine learning to predict AID targets

The conditional inference tree for classification was built using the *ctree* function from the party R package with default parameters. Genes with a background mutation frequency >5 × 10^−4^ were excluded to avoid artifacts. The following variables were fed into the model for each of the 1,339 genes analyzed: expression, transcription rate, PolII recruitment, and Spt5 recruitment (quantitative, continuous); Med12 recruitment, H3K4me1 recruitment, H3K36me3 recruitment, H3K79me2 recruitment, regulation by superenhancers, and occurrence of convergent transcription (qualitative, discrete). All variables were assigned equal weights to fit the model.

#### Annotation of AID targets

Annotation of AID targets was performed based on public data on sequencing of human DLBCLs, Burkitt lymphomas, and follicular lymphoma tumors (Lohr et al., 2012; Love et al., 2012; Morin et al., 2013; Zhang et al., 2013; de Miranda et al., 2014; Okosun et al., 2014).

### Data availability

Sequencing data generated for this study are available through the GEO database: targeted DNA deep sequencing (accession no. GSE102944) and RNA-Seq (accession no. GSE98086).

The rest of the datasets analyzed in the current study are publicly available through the GEO and/or Sequence Read Archive: GRO-Seq (accession no. GSE62296), GC B cells (accession nos. SRR1611832, SRR1611833, and SRR1611834), naive B cells (accession nos. SRR1611829, SRR1611830, and SRR1611831), ChIP-Seq of PolII and Spt5 (accession no. GSE24178), and ChIP-Seq data of epigenetic marks Med12 (accession no. SRX347810), H3K4me1 (accession no. SRX347815), H3K36me3 (accession no. SRX185869), and H3K79me2 (accession no. SRX185843).

### Statistical analysis

Statistical analyses were performed with stats R package v3.1.1. Error bars in figures represent SEM. Student’s *t* test was applied to continuous data, and a Fisher test was used to assess differences between categorical variables. P-values were corrected for multiple hypothesis testing by Benjamini-Hochberg or Bonferroni method where appropriate. Differences were considered statistically significant at P ≤ 0.05 or q ≤ 0.05.

### Online supplemental material

Fig. S1 shows the experimental workflow used to identify AID targets and technical controls. Fig. S2 shows mutation analysis of WRCY/RGYW hotspots. Fig. S3 shows details on the machine-learning classification tree used for the prediction of AID targets. Table S1 contains a list of the genes included in the capture library. Table S2 A contains a detailed mutation analysis of AID targets in *Ung^+/−^Msh2^+/−^*, *Ung^−/−^Msh2^+/−^*, *Ung^+/−^Msh2^−/−^*, and *Ung^−/−^Msh2^−/−^*. Table S2 B contains a list of the 18 AID targets mutated in repair-proficient GC B cells. Table S3 shows mutation analysis of genes validated by Sanger sequencing. Table S4 shows mutation analysis of the genes selected for machine-learning validation. Table S5 contains a list of the mutations found in *Ung^−/−^Msh2^−/−^* GC B cells that have been identified in cohorts of human lymphoma patients.

## Supplementary Material

Supplemental Material

Tables S1-S5 (zipped Excel files)
