# Supplementary material for: A broad atlas of somatic hypermutation allows prediction of activation-induced deaminase targets
Source: J Exp Med. 2018 Mar 5;215(3):761–71. doi: 10.1084/jem.20171738 (PMC5839764; doi:10.1084/jem.20171738)
Supplement: Supplemental Material [file JEM_20171738_sm.pdf]

Journal of Experimental Medicine

SUPPLEMENTAL MATERIAL

Álvarez-Prado et al., https://doi.org/10.1084/jem.20171738

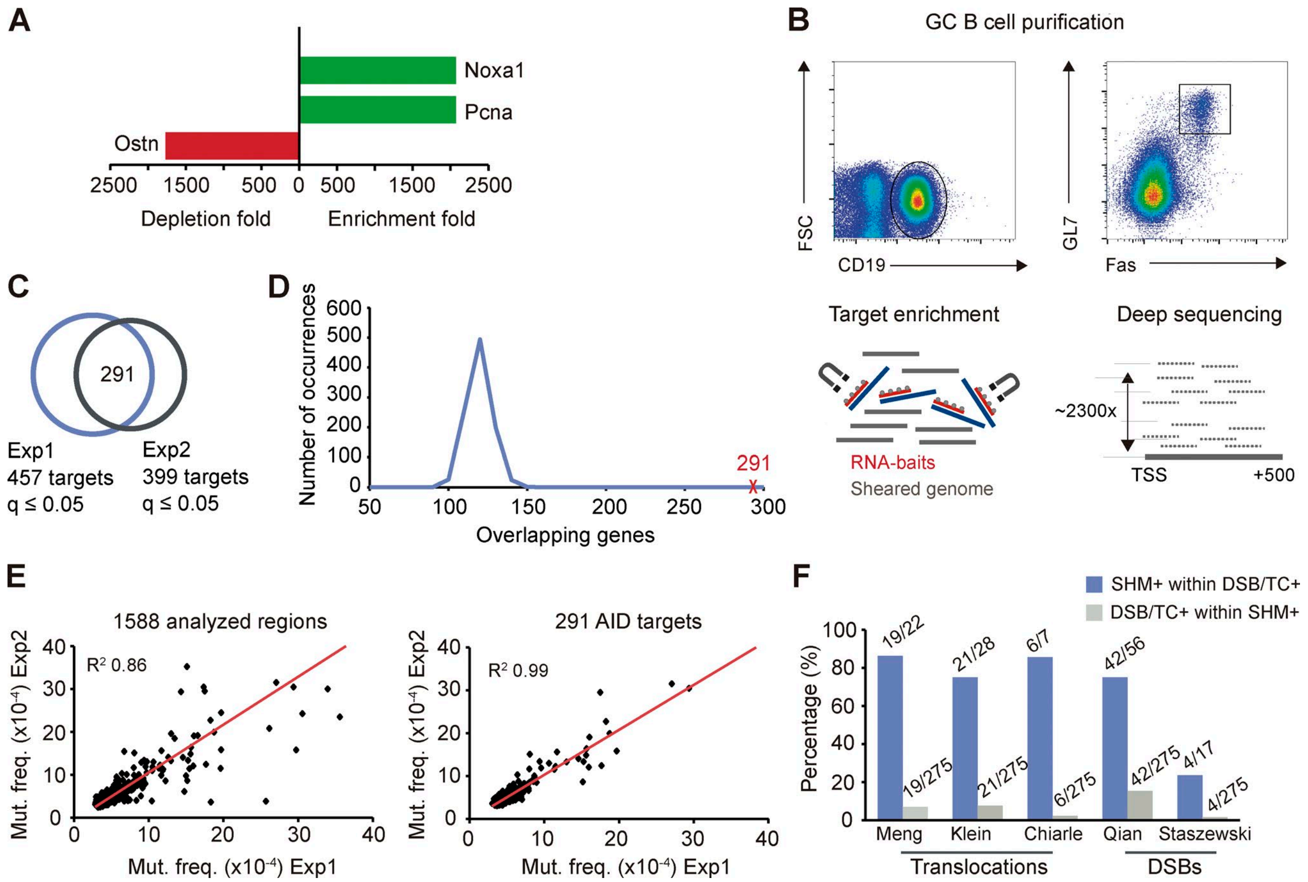

Figure S1. **Identification of AID targets by target enrichment coupled to next generation sequencing. (A)** Target enrichment protocol allows a 2,000-fold enrichment of selected genes. Genomic DNA corresponding to genes included (*Noxa1* and *PCNA*) and not included (*Ostn*) in the SureSelect capture library was quantified by quantitative RT-PCR before and after DNA capture enrichment. Graph represents fold depletion or fold enrichment calculated as $2^{(C_T\text{Input}-C_T\text{Enriched fraction})}$. Mean of two independent experiments is represented. **(B)** Schematic representation of the experimental approach used. GC (*CD19⁺Fas⁺GL7⁺*) B cells from Peyer's patches were isolated by cell sorting, and genomic DNA was extracted, sheared, and captured with a custom library of RNA probes. Enriched DNA was subjected to next generation sequencing to achieve a mean depth of 2,300 reads per nucleotide. **(C)** Two independent experiments were performed (Table S2) with 457 mutated targets found in Exp1 and 399 in Exp2. An overlap of 291 AID targets was found between Exp1 and Exp2. **(D)** Experimental distribution of random overlaps simulated for 1,000 iterations. For each iteration, random groups of 457 and 399 genes were selected from the genes included in the SureSelect capture library, overlapped, and the number of coincident genes reported. The probability to find an overlap of 291 genes by chance is <1 out of each 10^{16} times tested. Two-tailed Fisher test; $P = \sim10^{-16}$. **(E)** Mutation frequencies of the 1,588 TSS proximal regions analyzed and the 291 targets found in two independent experiments. **(F)** Percentage of genes undergoing DSB/TC+ according to the indicated studies within AID mutational targets described in this study (SHM+; 275 genes obtained in two independent experiments) and percentage of SHM+ genes within DSB/TC+ genes (see Materials and methods).

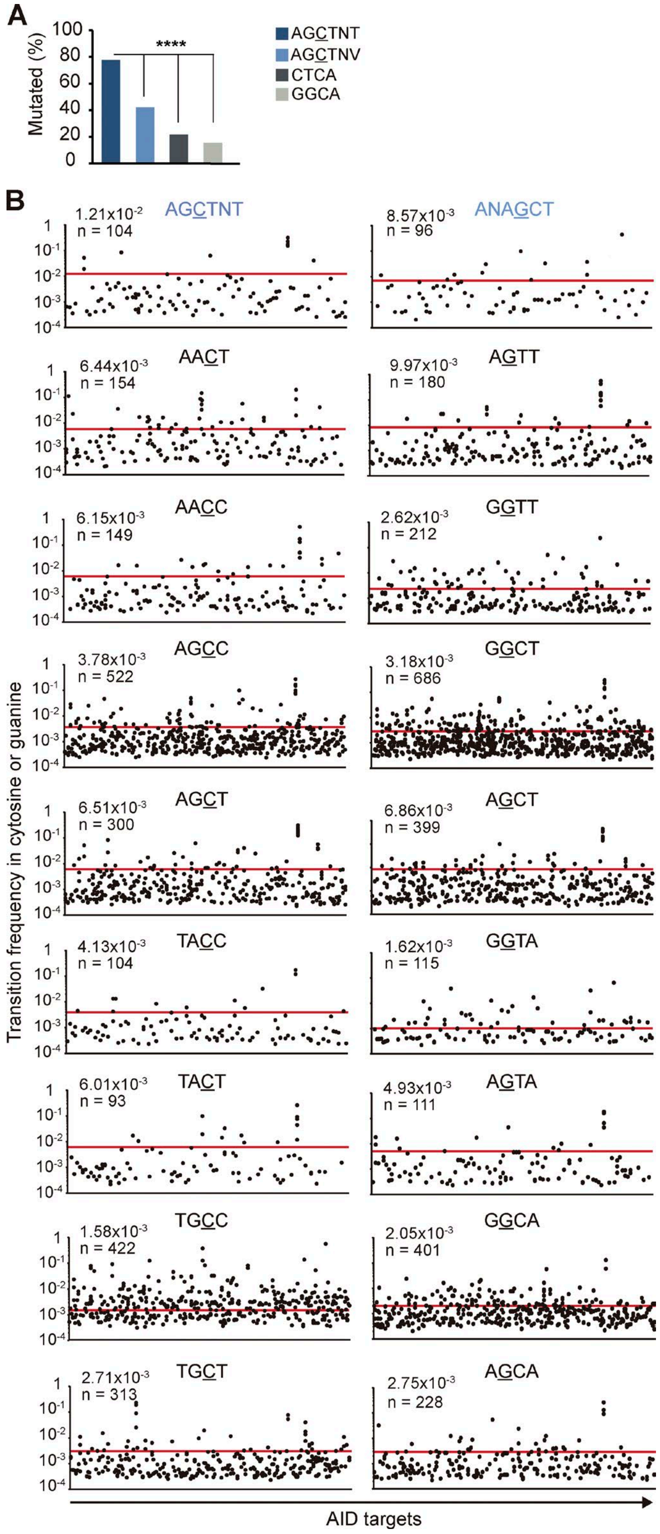

Figure S2. **Mutation analysis at WRCY/RGYW hotspots in *Ung*$^{-/-}$*Msh2*$^{-/-}$ GC B cells. (A)** Percentage of mutated cytosines within AGCTNT and AGCTNV hotspots and CTCA and GGCA non–hotspot motifs (Fisher test; ****, $P < 10^{-13}$). **(B)** Plots show mutated individual hotspots (WRCY, left; RGYW, right). Newly identified AGCTNT/ANAGCT hotspots are shown in the top row. Within each plot, each dot represents an individual WRCY/RGYW motif found mutated at least once. Each position in the x axis corresponds to a different gene, and the y axis shows mutation frequency of each individual hotspot within a gene. Mean mutation frequency is indicated and depicted with a red line. Number of mutated hotspots is indicated.

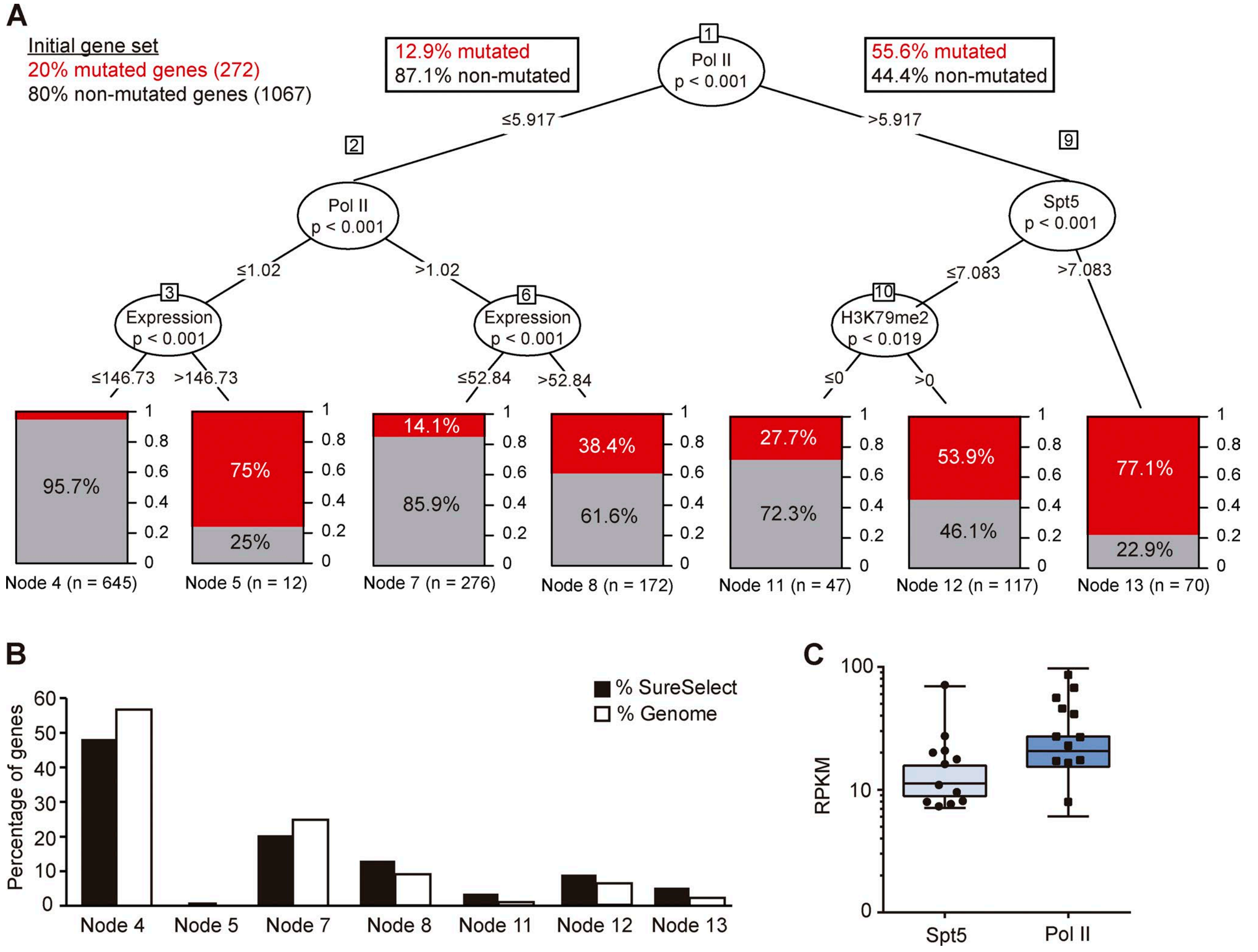

Figure S3. **Machine learning to predict AID targets genome wide. (A)** Recursive partitioning tree model classifies AID targets based on different molecular features: mRNA expression, PolII and Spt5 recruitment, and presence of H3K79me2 epigenetic mark (see Materials and methods). Each node splits the genes into two significantly different groups based on a particular feature. Numbers within the branches indicate the thresholds used to make the groups; p-values of each decision are included below the parameter measured in each node. **(B)** Bar graph depicting the proportion of SureSelect genes (1,339 genes; closed bars) or of total genes in the mouse genome (17,858 genes; open bars) that meet the thresholds established in each node. **(C)** Box plot depicting genome-wide data of PolII and Spt5 recruitment in in vitro activated B cells. Black dots and squares mark the 12 genes selected for the validation of the model prediction. RPKM, reads per kilobase per million reads mapped.

Tables S1–S5 are provided in separate Excel files.

Table S1 contains a list of the genes included in the capture library.

Table S2 A contains a detailed mutation analysis of AID targets in *Ung$^{+/-}$Msh2$^{+/-}$*, *Ung$^{-/-}$Msh2$^{+/-}$*, *Ung$^{+/-}$Msh2$^{-/-}$*, and *Ung$^{-/-}$Msh2$^{-/-}$*. Table S2 B contains a list of the 18 AID targets mutated in repair-proficient GC B cells.

Table S3 shows mutation analysis of genes validated by Sanger sequencing.

Table S4 shows mutation analysis of the genes selected for machine-learning validation.

Table S5 contains a list of the mutations found in *Ung$^{-/-}$Msh2$^{-/-}$* GC B cells that have been identified in cohorts of human lymphoma patients.
